# Comparative analysis of signature genes in PRRSV-infected porcine monocyte-derived cells to different stimuli

**DOI:** 10.1371/journal.pone.0181256

**Published:** 2017-07-20

**Authors:** Laura C. Miller, Damarius S. Fleming, Xiangdong Li, Darrell O. Bayles, Frank Blecha, Yongming Sang

**Affiliations:** 1 Virus and Prion Diseases of Livestock Research Unit, National Animal Disease Center, USDA-ARS, 1920 Dayton Ave, Ames, United States of America; 2 National Research Center for Veterinary Medicine, Cuiwei Rd, High-Tech District, Luoyang, Henan, China; 3 Infectious Bacterial Diseases Research Unit, National Animal Disease Center, USDA-ARS, 1920 Dayton Ave, Ames, United States of America; 4 Department of Anatomy and Physiology, College of Veterinary Medicine, Kansas State University, Manhattan, United States of America; 5 Department of Agricultural and Environmental Sciences, College of Agriculture, Human and Natural Sciences, Tennessee State University, 3500 John A. Merritt Boulevard, Nashville, United States of America; Emory University School of Medicine, UNITED STATES

## Abstract

Monocyte-derived DCs (mDCs) are major target cells in porcine reproductive and respiratory syndrome virus (PRRSV) pathogenesis; however, the plasticity of mDCs in response to activation stimuli and PRRSV infection remains unstudied. In this study, we polarized mDCs, and applied genome-wide transcriptomic analysis and predicted protein-protein interaction networks to compare signature genes involved in mDCs activation and response to PRRSV infection. Porcine mDCs were polarized with mediators for 30 hours, then mock-infected, infected with PRRSV strain VR2332, or a highly pathogenic PRRSV strain (rJXwn06), for 5 h. Total RNA was extracted and used to construct sequencing libraries for RNA-Seq. Comparisons were made between each polarized and unpolarized group (i.e. mediator vs. PBS), and between PRRSV-infected and uninfected cells stimulated with the same mediator. Differentially expressed genes (DEG) from the comparisons were used for prediction of interaction networks affected by the viruses and mediators. The results showed that PRRSV infection inhibited M1-prone immune activity, downregulated genes, predicted network interactions related to cellular integrity, and inflammatory signaling in favor of M2 activity. Additionally, the number of DEG and predicted network interactions stimulated in HP-PRRSV infected mDCs was superior to the VR-2332 infected mDCs and conformed with HP-PRRSV pathogenicity.

## Introduction

The status of the host immune system during viral infections is an important key to uncovering more efficient means of tackling disease among porcine livestock. An important portion of untangling the host immune response is first gaining insight on the stimulatory effects that various immune molecules such as monocyte derived cells (mDCs) like macrophages (MΦs) and dendritic cells (DCs) play in antiviral immunity [1–6]. For the most part, this information is still unknown in regards to porcine monocyte derived cells (mDCs) and their reaction to infections by economically important monocytotropic porcine viruses (attacking various monocytic cells) including our focus, porcine reproductive and respiratory syndrome virus (PRRSV). Understanding the relationship of mDC stimulation and immune response as stated by Sang et al., 2014, “not only extends the activation paradigm of these cells” but, it will also help to integrate innate immune responses with “aspects of inflammation, tissue repair and overall antimicrobial activity” [4]. This is important because most PRRSV infections are syndromes complicated with co-infection from pathogens of other phyla. Therefore, integration of conventional activation status and antiviral states provides a framework for potentiation of the overall immune response to PRRS disease. Because of the ability of PRRSV to infect mDCs [7,8] and subvert immune responses [6,7,9,10] it is an archetype for investigating the interaction of immune cell activation statuses with host antiviral immunity.

MΦ activation statuses are classified as: interferon-gamma (IFN-γ) classically activated macrophages (M1), interleukin-4 and 13 (IL-4/IL-13) alternatively activated macrophages (M2), and interleukin-10 (IL-10) deactivated macrophages (dM) [2,11–13]. We have previously shown that porcine monocytic-derived cells at different activation status are permissive and respond differentially to PRRSV infection [4,6]. The M2 activation statuses, including IL-10 and IL-4 activated cells, are highly permissive to PRRSV infection compared to the M1 statuses induced by IFN-γ and LPS regarding their inflammatory and immune signaling functions [6]. Of the mediators, the interferon-alpha (IFN-α) induced antiviral state exerts the highest virostatic activity; however, the interferon-beta (IFNβ) induced state has much less antiviral activity against PRRSV in macrophages and have shown to be less virostatic than the M1 stimulated cells [6].

In order to study how PRRSV infection alters cell activation, we have systematically characterized mDC activation status and determined genome-wide differential gene expression regulating porcine mDCs infected with PRRSV using our established RNA-Seq procedure [4–6]. This comparison also allowed us to determine significant gene responsive pathways shared between mDCs and macrophages.

## Materials and methods

### Cells, virus infection and RNA-seq analysis

Porcine mDCs were polarized with mediators (PBS, IFN-γ, IL-4, LPS, IL-10, IFN-α) for 30 hours, then mock-infected, infected with PRRSV strain VR2332, or highly pathogenic strain rJXwn06 (HP-PRRSV), for 5 h. The mDCs pooled for RNA extraction were derived from blood monocytes from at least five outbreed piglets that have similar genetic background. Our pooled design is to represent typical outbreed piglets rather than profiling individual biological difference. The mDCs pooled for RNA extraction were derived from blood monocytes (10^8^ cells/sample) from at least five outbreed piglets that have similar genetic background. Culturing of monocytes to produce mDCs occurred through use of IL-4 and granulocyte-macrophage colony-stimulating factor (GM-CSF) for 7 days as previously described in Sang et al., 2014 [4,6]. Porcine mDCs were polarized and infected with PRRSV strain VR2332, or highly pathogenic strain rJXwn06 (HP-PRRSV), at a multiplicity of infection (MOI) of 0.1 TCID_50_/ml for 5 h and washed twice with fresh culture medium prior to RNA and protein extraction [6]. All mediators (purchased from R&D Systems, Minneapolis, MN, or Sigma-Aldrich, St. Louis, MO) were dissolved in 1× Dulbecco's phosphate-buffered saline (DPBS) (Invitrogen) containing 1% bovine serum albumin (BSA) (fraction V, cold ethanol precipitated; Sigma) and applied (1:100) to the cultured cells at 20 ng/ml described in Sang et al., 2014 [4,6]. Total RNA was extracted from the pooled cells of four replicates, and used to construct sequencing libraries for the transcriptomic analysis. Sequence libraries were used to generate 100 bp paired-end reads using the Ilumina® 2500 HiSeq. Procedures for read processing, mapping, and alignment previously described and optimized in Miller et al., 2012 [14]. The data from the samples was normalized, with respect to library size, to compare changes in expression between the paired comparisons (Table 1), resulting in thirty-three comparison tables overall. After normalization, a heat map highlighting the 35 most variable genes from the expression analysis was generated based upon read counts. In this plot, we did not do any comparison of the differential expression; rather, we simply looked at read counts for each gene across all treatments to examine which genes showed the most variability. The logic is that the most variable genes are likely to be the genes that will provide the most resolution for clustering the samples. Normalization of gene counts was carried out using the rlog transformation function and calculations of differential gene expression for each comparison were based upon the average log expression (avgLogExpr) and the regularized log2 fold change (rLogFC) using DESeq2 [15]. Significant differential gene expression was based on the rLogFC in expression and within-group variability (dispersion). The dispersion estimates for the genes were acquired based on data for each individual gene. Comparisons of the change in expression employed the rlog transformed average of genes across samples to a log2 scale to incorporate genes for which evidence of strong fold changes was weak due to low counts. Once that transformation was applied the avgLogExpr and rLogFC were calculated. The data were reanalyzed without being sized for more sensitivity for the 2 samples. Genes were annotated using the Ensembl database [16] and gProfiler [17] then ranked based upon the DESeq2 [15] calculated rLogFC to determine rank genes according to their expression difference between the samples. The rLogFC analysis does not generate a p-value cut-off. Downstream analysis of the differential expression of genes from the comparisons used a rLogFC threshold of ≥ 2 and ≥ -2 to designate expression as being down or upregulated.

**Table 1:** Sample organization table.

CTRL Grp I:	Polarization mediators	PRRSV (moi: 0.1)
1	PBS	N/A
2	IFN-γ	N/A
3	IL-4	N/A
4	LPS	N/A
5	IL-10	N/A
6	IFN-α	N/A
VR Grp II:	Polarization mediators	PRRSV (moi: 0.1)
1V	PBS	VR-2332
2V	IFN-γ	VR-2332
3V	IL-4	VR-2332
4V	LPS	VR-2332
5V	IL-10	VR-2332
6V	IFN-α	VR-2332
HP Grp III:	Polarization mediators	PRRSV (moi: 0.1)
1H	PBS	HP-JX
2H	IFN-γ	HP-JX
3H	IL-4	HP-JX
4H	LPS	HP-JX
5H	IL-10	HP-JX
6H	IFN-α	HP-JX

Comparisons were made within each treatment group of activation status (mediator vs. PBS control) and between treatment group for each mediator. Sample controls consisted of the CTRL Grp I (1-PBS), VR Grp II (1V-PBS-VR-2332), and HP Grp III (1H-PBS-HP-JX).

### Comparison of gene expression based on mediator stimulation

The ability of the 5 mediators to stimulate classical M1 or M2-like differential gene expression in mDCs during PRRSV pathogenesis was investigated across all treatments to generate 33 gene lists (Table 2) based on the Table 1 comparisons. The controls (comparisons #1–5) were used to observe the overall action of the mediator on mDCs in their “natural” state. The activity of the mediators was also observed in the VR-2332 and HP-PRRSV infected mDCs (comparisons # 6–15). Comparisons #17–27 allowed observation of the effect viral infection would have on the mDCs after 30-hrs of mediator polarization. The last five comparisons (#28–33) examined the mediator effect on VR-2332 infected mDCs vs. HP-PRRSV infected mDCs. (S1 Table).

**Table 2:** List of sample comparisons.

IFN-γ	2	vs	1	comp1
IL-4	3	vs	1	comp2
LPS	4	vs	1	comp3
IL-10	5	vs	1	comp4
IFN-α	6	vs	1	comp5
IFN-γ	2V	vs	1V	comp6
IL-4	3V	vs	1V	comp7
LPS	4V	vs	1V	comp8
IL-10	5V	vs	1V	comp9
IFN-α	6V	vs	1V	comp10
IFN-γ	2HP-JX	vs	1HP-JX	comp11
IL-4	3HP-JX	vs	1HP-JX	comp12
LPS	4HP-JX	vs	1HP-JX	comp13
IL-10	5HP-JX	vs	1HP-JX	comp14
IFN-α	6HP-JX	vs	1HP-JX	comp15
PBS	1V	vs	1	comp16
IFN-γ	2V	vs	2	comp17
IL-4	3V	vs	3	comp18
LPS	4V	vs	4	comp19
IL-10	5V	vs	5	comp20
IFN-α	6V	vs	6	comp21
PBS	1HP-JX	vs	1	comp22
IFN-γ	2HP-JX	vs	2	comp23
IL-4	3HP-JX	vs	3	comp24
LPS	4HP-JX	vs	4	comp25
IL-10	5HP-JX	vs	5	comp26
IFN-α	6HP-JX	vs	6	comp27
PBS	1HP-JX	vs	1V	comp28
IFN-γ	2HP-JX	vs	2V	comp29
IL-4	3HP-JX	vs	3V	comp30
LPS	4HP-JX	vs	4V	comp31
IL-10	5HP-JX	vs	5V	comp32
IFN-α	6HP-JX	vs	6V	comp33

Thirty-three comparisons were made from the data between each treatment group (control, VR-2332, and HP-PRRSV) and each mediator listed in Table 1. The group numbers from Table 1 correspond to the samples and treatments contrasted within each of the 33 comparisons. These comparisons were used to generate the differentially expressed genes used for downstream analysis. Comparisons 1–15: each mediator (to the PBS treatment. Comparisons 16–33: infected (V, HP-JX) with uninfected, and infected (V) with infected (HP-JX).

### Predicted gene interaction networks

In order to further examine the possible pathogenic effects and differences the mediators had on the VR-2332 and HP-PRRSV infected mDCs, a list of differentially expressed genes with a regularized log2 fold change (rLogFC) ≥ 2 or ≥ -2 was supplied from each comparison to the program STITCH (versions 4.0 and 5.0) [18,19]. The programs were used to predict any potential protein-protein/chemical interactions that may be common or unique to the different combinations of PRRSV infection and stimuli, in order to provide a visual output of the molecular actions taking place within the generated networks. The software accomplishes this through the input of the generated RNA-seq gene list without expression values (query list), that is then compared against multiple curated databases to evaluate and predict possible network interactions displayed as nodes (genes) and edges (line connecting nodes). The software does not differentiate between the expression levels of the genes in the list, but does draw in information from various databases to predict the effect (positive, negative, unknown) a gene is expected to exert on another in the network. The parameters used to generate the networks within the software consisted of: use of all evidence databases described by STITCH [18,19], view settings were fixed to display all predicted network/interactions based upon their “molecular action”, a confidence score threshold of ≥ 0.9 to identify an interaction, and the maximum number of allowed predictors (i.e. external genes) was ten. Use of the molecular action setting gives an output in which the color and shape of the lines (edges) can be used to denote the mode of action. In the predicted network outputs, a red line connecting nodes represents inhibition, green lines represent activation; dark blue lines represent binding; purple lines represent catalysis; yellow lines represent transcriptional regulation, light blue represents phenotype, and black lines are representative of reaction. Greyed-out edges represent predicted interactions in which the mode of action is unknown. The shape of the edge also denoted the predicted effect (action) of a node on another connected by the edge [18,19]. All annotations were based upon the Sscrofa10.2 reference genome. The results from the predicted networks are grouped by the gene mediator (PBS, IFN-γ, IL-4, LPS, IL-10, or IFN-α) present within each comparison. Any comparison without genes with a fold change ≥ +/- 2 (comparison 19) or differentially expressed genes that could not be annotated due to them being an amalgam of novel genes, micro-RNA, ribosomal genes, and small nuclear genes (comparisons 13, 23, and 26) was not explored. The resulted network images mainly display those genes (query and allowed predictors) that formed networks due to size limitations and to increase figure readability. All genes used in the query are available in supplementary S1 Table.

The trends in read count variation, differential gene expression between treatments, and the network interactions predicted from the comparisons are treated as exploratory in nature for the screening of candidate genes involved in the host response to PRRSV pathogenesis for further study.

## Results

### Gene variation among infections and mediators

**Fig 1:**
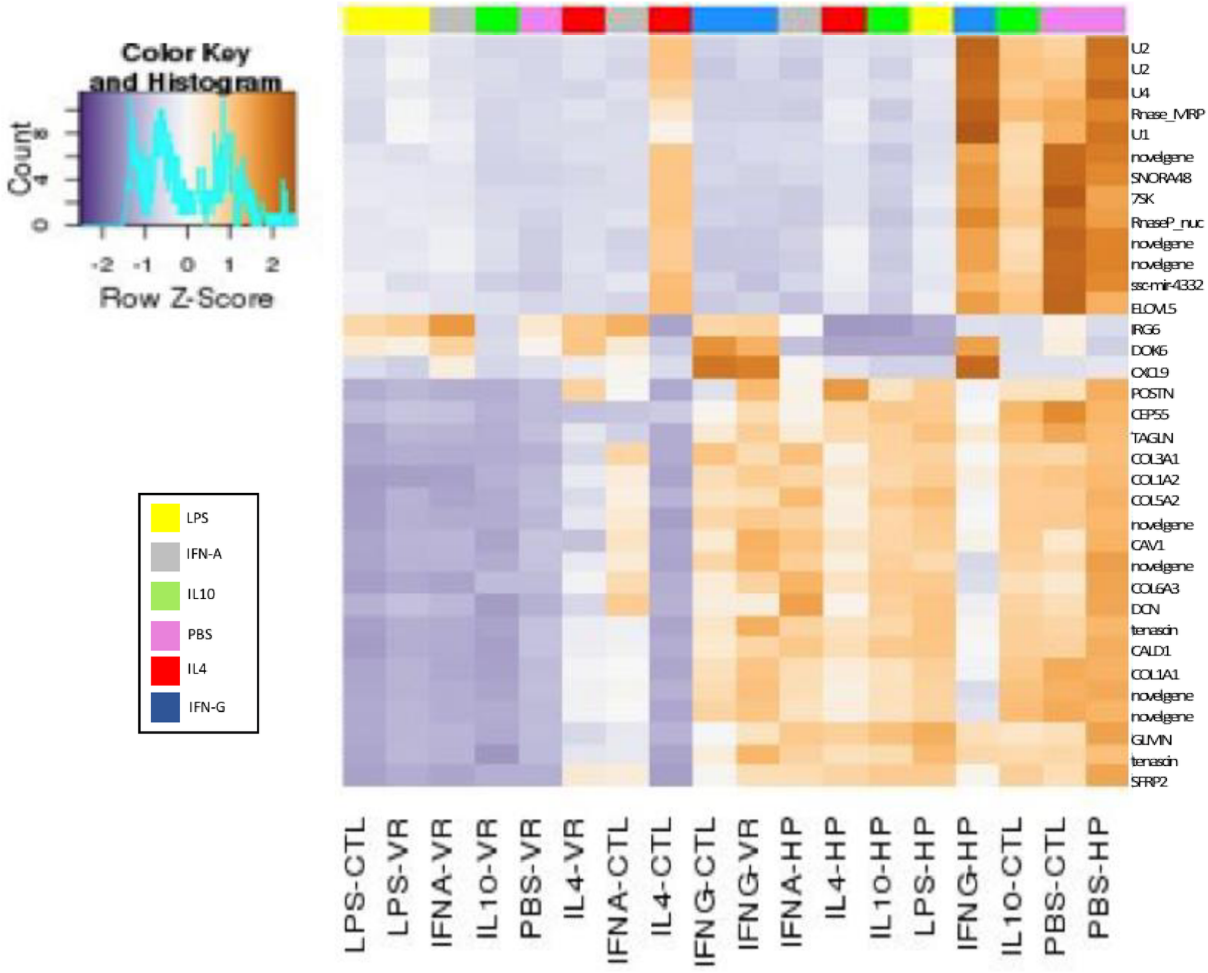
Heat map of the top 35 genes with the most variable read counts in the dataset. The bar along the top of the plot highlights the mediators in which the gene counts varied. This is also reinforced in the names on the x-axis, which show the treatment and mediator in which the genes (Y-axis) showed high variation. CTL = Control samples; VR = VR-2332 PRRSV strain; HP = High pathogenic strain rJXwn06. The plot showed possible candidates for the genes that appear to impact PRRSV pathogenesis based upon the contrasts. One particular gene from this list, the proteoglycan *DCN*, was also show to be highly variable across treatments based upon normalized fold change. The gene *DCN* also appeared multiple times in the predicted networks analysis as part of a group of differentially expressed proteoglycans shown to be related damage associated molecular pattern signaling.

The heat map (Fig 1) showed that for the 35 genes listed VR-2332 and HP-PRRS have different effects on the number of gene reads vs. the control (CTL), which can inform about “trends” among the samples. The gene names, which have been converted from Ensembl [16] identifiers, are on the y-axis of Fig 1. Contained within this list of 35 are the short non-coding (snc) poly A and spliceosomal RNA U1, U2, U4, RNase for mitochondrial processing (Rnase-MRP), small nucleolar RNA (snora48), nuclear RNase P (RnaseP_nuc) and microRNA (ssc-mir-4332). A number of cellular component immune signaling genes were also among the list of most variable. These genes included elongation of very long fatty acids protein 5 (*ELOVL5*), docking platform for assembly of multimolecular signaling complexes (*DOK6*), periostin ligand for α_v_β_3_ and α_v_β_5_ integrins (*POSTN*), centrosomal protein of 55kDa (*CEP55*) which has a role in the mitotic exit and cytokinesis, transgelin (*TAGLN*) whose downregulation signals the onset of transformation. Also within this list were multiple collagen genes (*COL3A1*, *COL1A2*, *COL5A2*, *COL6A3*, *COL1A1*), caveolin scaffolding protein (*CAV1*), decorin (*DCN*) and tenascin (*TNN*), genes known to be components of connective tissue of the extracellular matrix or involved in heparin or collagen binding and PI3K-Akt signaling. Additionally in the list were the genes caldesmon1 (*CALD1*)–a calmodulin and actin binding protein involved in integrin and cytoskeletal signaling pathways, glomulin (*GLMN*) which is involved in ubiquitin protease ligase binding and hepatocyte growth factor binding, and soluble frizzled-related proteins 2 (*SFRP2*)–a modulator of Wnt signaling [16,20]. The antiviral genes amongst the 35 most variable genes by read count are inflammatory response protein 6 (*IRG6*) shown to be highly expressed in HP-PRRSV infection [10,21], and antiviral chemokine ligand 9 *(CXCL9*) induced by interferon-γ involved T-cell trafficking, signaling by G protein-coupled receptors, and Akt-signaling [16,20]. Many of the genes listed in Fig 1 also appear as differential expression in the sample comparisons (S1 Table).

### Differential gene expression from contrasts

#### Comparisons #1–5 mediator stimulation of naïve mDCs

In the presence of sham inoculation (comparisons #1–5), the M1/M2 mediators LPS, IL-4, and IFN-γ were able to elicit upregulation of the immune function related genes such as *IRG6* (*IL-4*, LPS) and *POSTN* (*IFN-γ*, LPS). The overall trend was one of upregulation of gene expression for the effects of the mediators on the naïve mDCs. Despite this trend for the gene lists, the mediators IFN-γ and IFN-α exhibited reduced expression of pro-inflammatory genes including *CXCL9*. Also, the mediators LPS and IL-4 elicited immune gene expression and were also shown to stimulate up regulation of a group of proteoglycans that possess both structural integrity and immune signaling capabilities.

#### Comparisons #6–15 mediator stimulated infected mDCs

**Fig 2:**
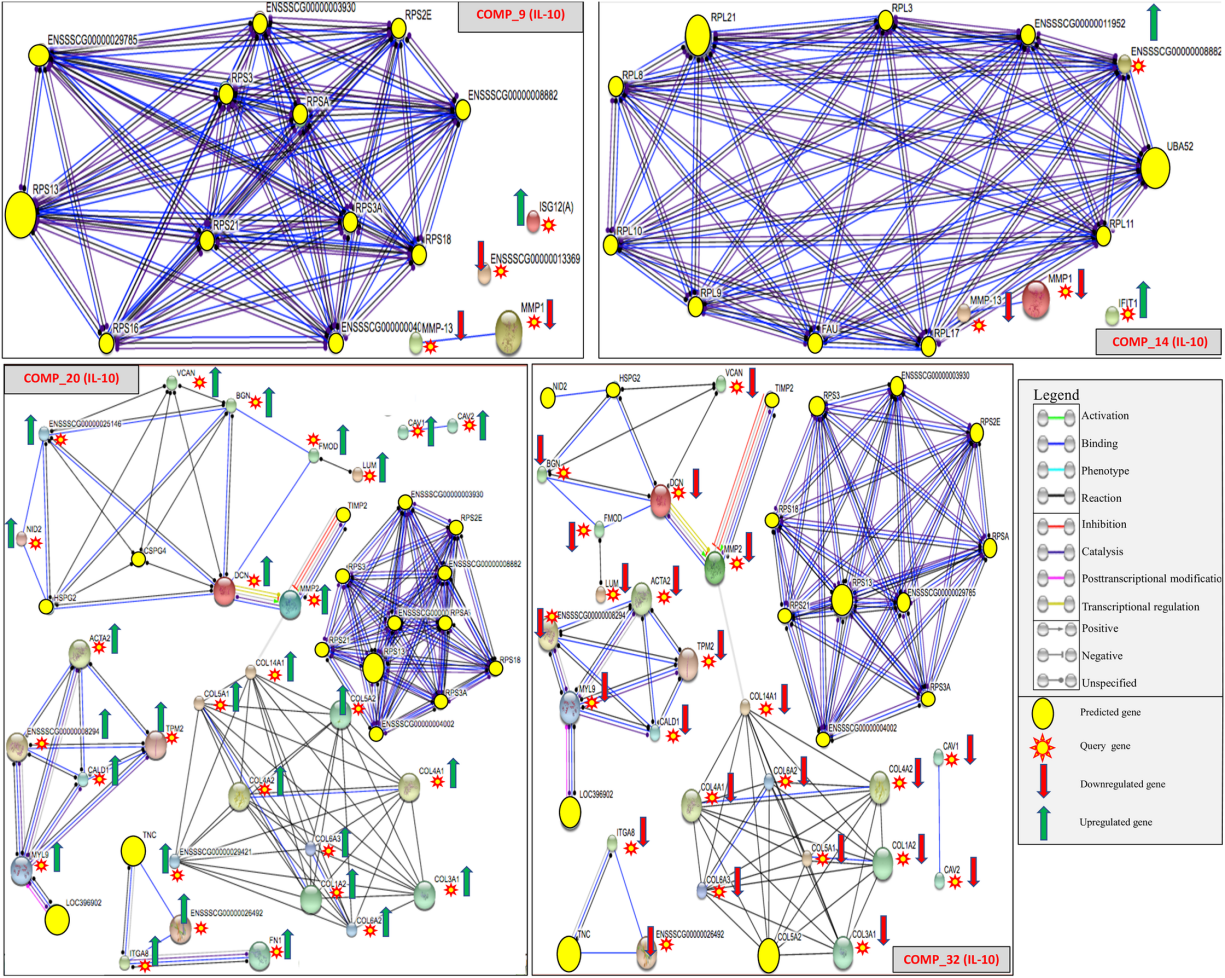
Predicted protein-protein and protein-chemical interactions for comparisons involving IL-10. Comp_4 shows the effect IL-10 has on non-infected mDCs. Comparisons using IL-10 mediators for VR-2332 and HP-PRRSV infected mDCs showed no proteoglycan network stimulation (Comp_9 and Comp_14 respectively). Predicted protein-protein and protein-chemical interactions for comparisons #20 and #32 displayed interactions from the predicted proteoglycan network. These genes, *BGN*, *DCN*, and *VCAN*, also function as damage associated molecular pattern signaling (DAMPs) genes. The genes, *BGN*, *DCN*, and *VCAN*, have additional immune actions as pro-inflammatory DAMPs that signal through pattern recognition receptor (PRR) cell types to initiate a cytokine response. Gene (nodes) represented by circles and the color of the connecting lines (edges) defines the molecular action of the connected nodes. Direction of expression based on rlogFC threshold of ≥ -2 or 2.

For these comparisons, the mDCs were infected with either VR-2332 (comparisons #6–10) or HP-PRRSV (comparisons #11–15) prior to stimulation with the mediators to observe expression changes. This set of comparisons was used to examine if any of the mediators could stimulate the mDCs to mount an antiviral response and observe if that response was more M1 or M2-like. We observed that the mediators had different degrees of stimulatory action on the mDC’s dependent on whether VR-2332 or HP-PRRSV was present. When examined, the VR-2332 infected comparisons (#6–10) had lower numbers of DE genes and showed an overall trend of downregulation for immune genes across the comparisons. Only IL-10 (Fig 2) and IFN-α showed any upregulation of genes with immune function via the genes interferon, alpha-inducible protein 27 (*ISG12(A*)) and thrombospondin 1(*THBS1*) respectively. Although *THBS1* was upregulated, it is also known to be a negative regulator of dendritic cells [16,20] and therefore may work in the virus’ favor during PRRSV infections. Comparisons #11–15 examined the HP-PRRSV infected mDCs and displayed an opposite trend from VR-2332, with most genes showing upregulation after stimulation. However, most of these genes were annotated to non-coding RNAs that showed high variability across treatments based on read counts (Fig 1). In respect to genes with immune functions the mediators, except for LPS, showed a general trend of downregulation. A similar trend could also be observed in genes related to structural integrity. Interestingly, the comparison stimulated by IL-4 was the only one in this group to upregulate expression of the proteoglycan *DCN* and the gene amphiregulin (*AREG*), the latter being a part of the Th2 cytokine profile [16]. In general, comparisons #6–15 exhibited decreased expression of M1-like and increased expression of M2-like responses for both viral strains.

#### Comparisons #16–27 virus effect on mediator stimulated mDCs

**Fig 3:**
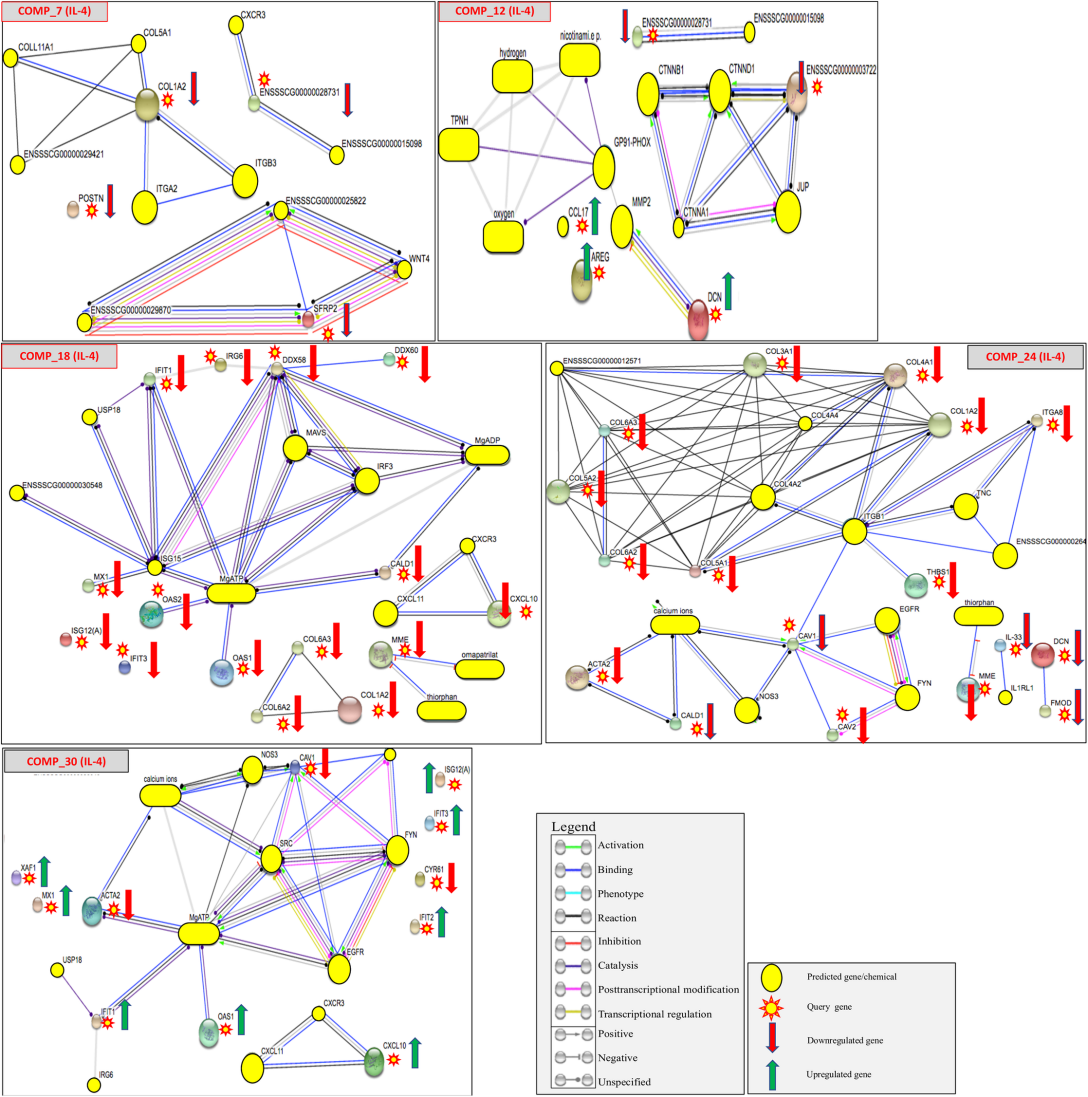
Predicted protein-protein and protein-chemical interactions for comparisons involving IL-4. The comparisons showed overall down regulation for both strains. The comparison infected with VR-2332 (Comp_7 and Comp_18) showed downregulation of more immune related genes and interactions, while the HP-PRRSV (Comp_12 and Comp_24) showed downregulation of more genes and networks related to structural integrity and immune signaling. Gene (nodes) represented by circles and the color of the connecting lines (edges) defines the molecular action of the connected nodes. Direction of expression based on rlogFC threshold of ≥ -2 or 2.

Infection of the IL-4 stimulated mDCs caused a trend of downregulation for both viruses, with the VR-2332 (comparisons #16–21) exhibiting mostly immune gene downregulation. This down-regulation was observed in the antiviral genes 2'-5'-oligoadenylate synthase 1 (*OAS1*), MX dynamin like GTPase 1(*MX1*), *IRG6*, *POSTN*, and a host of interferon induced protein with tetratricopeptide repeats (*IFIT*) genes. The effect of IL-10 (#20) on the VR-2332 infected mDCs was a stark contrast to IL-4, showing a large number of immune and structural genes to be upregulated (Fig 3). Overall the VR-2332 infection appeared to upregulate genes that favored the M2 response, as well as, causing upregulation of *DCN* and other proteoglycans against the various mediators.

In the HP-PRRSV infected mDCs (comparisons #22–27) none of the differentially expressed genes for IFN-γ and IL-10 had available annotations. In the remaining mediators, the majority of differentially expressed genes were downregulated. Much of the downregulation observed took place in genes with functions related to structural integrity, pattern recognition signaling, and response to cell damage (i.e. *CAV1*, *CALD1*, *DCN*, and multiple collagen genes) [20,22]. The HP-PRRSV infection appeared to also favor an M2 environment over the M1 mediators. Stimulation of the mDCs with IL-4 appeared to help downregulate genes with immune and structural functions (Table 3) (Fig 3). This trend was observed in most of the mediators except for IFN-α, which showed slight evidence of promoting an M1 response based on upregulation of *IRG6* and dowregulation of *AREG*.

**Table 3:** Candidate genes related to mediator stimuli and PRRSV infection.

	comparison #	mediator	prrsv strain	gene id	rlogfc
Comparisons #6–15 mediator stimulated infected mDCs	7	IL-4	VR-2332	*SFRP2*	-3.24
	9	IL-10	VR-2332	*ISG12(A)*	2.07
	12	IL-4	HP-PRRSV	*DCN*	2.11
	12	IL-4	HP-PRRSV	*AREG*	2.46
	14	IL-10	HP-PRRSV	*IFIT1*	2.01
Comparisons #16–27 virus action on mediator stimulated mDCs	18	IL-4	VR-2332	*IRG6*	-5.07
	18	IL-4	VR-2332	*OAS1*	-2.57
	18	IL-4	VR-2332	*IFIT3*	-3.26
	20	IL-10	VR-2332	*DCN*	5.67
	20	IL-10	VR-2332	*CAV1*	5.33
	20	IL-10	VR-2332	*FMOD*	3.86
	24	IL-4	HP-PRRSV	*DCN*	-2.55
	24	IL-4	HP-PRRSV	*FMOD*	-3.52
	24	IL-4	HP-PRRSV	*IL33*	-3.72
	24	IL-4	HP-PRRSV	*CALD1*	-4.27
Comparisons #28–33 mediator effect on VR-2332 vs. HP-PRRSV infected mDCs	30	IL-4	VR-2332/HP-PRRSV	*CXCL10*	5.57
	30	IL-4	VR-2332/HP-PRRSV	*ACTG2*	-3.60
	32	IL-10	VR-2332/HP-PRRSV	*VCAN*	-2.10
	32	IL-10	VR-2332/HP-PRRSV	*BGN*	-2.32
	32	IL-10	VR-2332/HP-PRRSV	*FMOD*	-3.85
	32	IL-10	VR-2332/HP-PRRSV	*DCN*	-5.47

Table shows genes from the IL-10 and IL-4 mediated comparisons. These comparisons showed transcriptomic responses that demonstrate the affinity for an M2-like environment during infection. Gene expression based on rlogFC threshold of ≥ -2 or 2.

#### Comparisons #28–33 mediator effect on VR-2332 infected mDCs vs. HP-PRRSV infected mDCs

The results from comparisons #28–33 were used to observe the differences between HP-PRRSV and VR-2332 infected mDCs by contrasting the two infections against each other for all mediators. This group of contrasts allowed observation of the magnitude of transcriptomic change in mDCs based on the differences in strength of the low or high pathonogenic PRRSV. The DEG results showed a general trend of gene downregulation across all of the mediators with the exception of IL-4, which showed a more a balanced response. The data showed that the IL-4 -stimulated mDCs generated an upregulated inflammatory response in the HP-PRRSV infected mDCs compared to VR-2332 infection (comparison #30) (Table 3). The HP-PRRSV caused an increased expression of *IRG6* and *OAS1*, genes known to have anti-viral, TLR, and pro-inflammatory signaling properties [23,24]. Upregulation of the interferon induced genes, *IRG6* and *OAS1*, may indicate that activation of these genes can also occur in the presence of IL-4, despite its anti-inflammatory functions. The gene *POSTN*, functions to signal chemokines in humans and mice [16,20], and was upregulated in the HP-PRRSV vs. VR-2332 infected comparison (#29) mediated by IFN-γ. The contrast examined for IL-10 (#32), showed the largest change in transcriptomic magnitude. In this comparison the detrimental effects of HP-PRRSV worsened in the presence of the mediator. Compared to VR-2332, HP-PRRSV in the presence of IL-10 -stimulated mDCs again downregulated multiple structural and immune genes (Table 3) that had been shown to be upregulated against VR-2332. This included *DCN*, biglycan (*BGN*), versican (*VCAN*), and fibromodullin (*FMOD*) a group of proteoglycan genes shown to have secondary immune functions [25,26].

### Prediction of protein-protein interaction networks

The genes with a differential expression ≥ +/- 2 rlogFC from the 33 comparisons (Table 2 and S1 Table) were used to predict possible protein-protein and protein-chemical interactions for all combinations of mediators and pathogens to examine networks possibly effected by the pathogen or the mediator based on the predicted interactions. The most common predicted gene-gene networks seen in the comparisons of PRRSV pathogenesis were related to structural gene networks. One of the most prevelant predicted interactions based on the DE genes was populated with a group of class I extracellular proteoglycans comprised of the genes biglycan (*BGN*), decorin (*DCN*), versican (*VCAN*), and fibromodullin (*FMOD*). These genes appeared downregulated in many of the comparisons that utilized HP-PRRSV as the infectious treatment and upregulated in those infected with VR-2332. These genes are of interest because they also have additional biological properties related to the host response to infection and are also known as damage associated molecular pattern signaling (DAMPs) genes [25,26]. The predicted proteoglycan interactions appeared in comparisons # 2, 3, 6, 16, 20, 21, 25, 28, and 31–33. The proteoglycan network was observed as extended networks that formed connections with immune and structural genes in the comparisons in which the HP-PRRSV isolate and/or IL-10 was used. This could be a possible indication of synergistic interaction of HP-PRRSV and the stimulatory cytokine. We also observed the appearance of partial proteoglycan networks in the presence of IL-4 stimulation. The four genes making up this network also appear as being variably expressed across many of the 33 comparisons utilizing the other mediators. The baseline PBS mediated comparisons predicted both the presence and expression of these genes in mock-infected and infected mDCs.

## Discussion

Differential gene expression of the samples was consistent with virus strain pathogenicity and mediator. Many of the genes that showed the most variability when infected with HP-PRRSV in the presence of M2 mediators were related to cellular structure and innate immune response. Additionally, the number of DEG and predicted network interactions stimulated in HP-PRRSV infected mDCs rivaled the VR-2332 infected mDCs and was in step with HP-PRRSV pathogenicity. The analysis of the mediator effect on the mDC response indicated that both VR-2332 and the HP-PRRSV inhibit M1 mediator activity (inflammation) and appear to prefer the M2 (anti-inflammatory) environment. This observation was echoed in the transcription profile which shows that, compared to the VR-2332 isolate, many of the DE genes were downregulated in the presence of the HP-PRRSV isolate regardless of the mediator. Though this is likely due to the difference in virulence between the isolates, mediator did have an effect as the number of HP-PRRSV downregulated genes tended to increase in the presence of M2 mediator activity. Substantial numbers of upregulated genes were observed when mDCs were sham inoculated (control) or infected by VR-2332 in conjunction with an M2 mediator such as IL-10. Overall, a trend of downregulation dominated the transcriptomic profile under infection, however, the genes C-X-C motif chemokine ligand 10 (*CXCL10*), *IRG6* and *CAV1* were upregulated for the majority of the comparisons. These genes represent candidates for further screening due to possessing molecular functions involved in anti-viral immune responses that may indicate or stimulate a pro-inflammatory environment. The gene *IRG6* represents a plausible candidate for further study because it is involved in interferon induced anti-viral immune functions, including cytokine production and Toll-like receptor (TLR) signaling [20], and has been shown to be significantly upregulated in previous studies of HP-PRRSV [10,21,27]. Caveolin 1 (*CAV1*), is a multifaceted immune function gene that targets viral lipids and is involved in TLR, Wnt, and apoptotic signaling, as well as, receptor mediated endocytosis of viruses [20,28]. Additionally, *IRG6* was upregulated in mDCs polarized with the IL-4 mediator, however, *CAV1* was downregulated. Despite this, the genes represent an overlap in anti-viral functions related to virus budding, replication, and virus-host matrix interactions possibly related to cell-surface receptor binding and signaling.

### Predicted networks indicate altered M1 activation of proteoglycan network in response to pathogen

The results show previously uncharacterized evidence of the ability of HP-PRRSV to effect M1 activation by altering expression of damage-associated molecular patterns (DAMPs) induced inflammation [25,26]. The genes at the center of the predicted *BGN-DCN-VCAN-FMOD* proteoglycan interaction network in Figs 2 and 3 were mostly downregulated and could be observed in ~38% (11/29) of the total comparisons and ~67% (4/6) of the HP-PRRSV rJXwn06 infected vs. VR-2332 infected group comparisons represented in Table 1. Proteoglycans are a group of structural proteins that are formed from a core protein and glycosaminoglicans (GAGs) and are found in extracellular matrices, connective tissue, and surface receptors [22,29–31]. The multitude of functions carried out by proteoglycans allow for their involvement in various immunological related processes such as DAMP recognition leading to TLR receptor signaling of pro-inflammatory mDCs. The appearance of the predicted proteoglycan network across multiple comparisons likely represented interference by PRRSV against possible activation of “stress” induced DAMP signaling during infection and/or replication. Damage-associated signals are based upon pattern recognition of physiological stress damage and not pathogen recognition like their counterpart, PAMPs [32,33]. In PRRSV infected swine these damage signals may be connected to the need for glycans [7,9,34] during viral receptor binding which might cause weakening of host structural matrix constituents (i.e. lipid binding proteins, collagen). Three of the genes, *BGN*, *DCN*, and *VCAN*, have been previously characterized in mice and humans as DAMPs because of their pro-inflammatory signaling ability through pattern recognition receptor (PRR) cell types which is a signaling ability similar to that of mDCs [6,8]. These genes are endogenous ligands of the extra-cellular matrix (ECM) that are induced by host stress (DAMP signals) to become soluble, act as ligands for the TLRs, and subsequently illicit an M1-like immune response of inflammation and autophagy [25,26,32,35]. Both *BGN* and *DCN* are small leucine-rich proteoglycans (SLRPs) with a core of leucine-rich repeats (LRRs) that interact with complementary TLR regions [22,25,35]. Versican, however, does not have this LRR region and is thought to bind and activate specific TLRs through its core protein [22,25]. Because the activation of this network of DAMPs leads to M1 innate immune responses, the down regulation of *BGN*, *DCN*, and *VCAN* during infection could effectively hinder the stimulation of the M1 (pro-inflammatory) mediators used in the current study.

The contrast of the mediators and infectious virions may indicate that PRRSV employs multiple methods to influence immune system effector activity through viral disruption, exploitation of cellular signaling, and structural integrity of monocytic derived cells. Perturbation of these multiple signaling pathways would be to the advantage of PRRSV replication. Additionally, evidence from the predicted network analysis indicated that this process is biologically further reaching and more destructive when cells are infected with the HP-PRRSV virion. This can be seen in the HP-PRRSV vs. VR-2332 (high virulence vs low virulence) comparisons which show the predicted *BGN-DCN-VCAN-FMOD* networks extended and connected to other predicted networks involved in structural matrices and immune responses. This extension, or linking, of the predicted proteoglycan network to others is a possible indication of monocytotrophic effects of PRRSV that perturbs multiple cell types and subsequently multiple biological processes or networks. Because these proteoglycans are able to cause sterile-inflammation (without pathogen recognition) based upon cell or tissue stress [25], it is possible that the predicted network represents a secondary signaling mechanism in healthy mDCs for initiating an innate immune response. This could possibly allow the host to mount an inflammatory response despite the adeptness of PRRSV to inhibit type I interferon mediated immune responses activated by the interferon mediators used in the current study. For the predicted local and extended *BGN-DCN-VCAN-FMOD* networks, *DCN* appears to act as a network hub, performing both binding and unspecified reaction functions with the other genes in the small multi-gene network. However, in the extended predicted *BGN-DCN-VCAN-FMOD* networks seen in the HP-PRRSV vs. VR-2332 (high virulence vs low virulence) comparisons (Figs 2 and 3), *DCN* extends its molecular action types (binding, reaction, activation, etc.) and becomes the hub for connecting the predicted proteoglycan interactions with larger predicted networks. These larger networks encompass differentially expressed genes with functions related to anti-viral activity such as, *CAV1* and structural integrity such as *TNN* and the multiple collagen structural genes (*COL3A1*, *COL1A2*, *COL5A2*, *COL6A3*, *COL1A1*) that were also highly differentially expressed. In the presence of the pathogen, this predicted network and others connected by the DCN hub, contained genes that were mostly downregulated indicating that PRRSV may be involved in subversion of both type I interferon [7,9] and ECM proteoglycan receptor signaling allowing PRRSV to better infect fibrous tissues such as alveolar and endometrial tissue [9,36]. The structural gene *DCN* in the predicted network was one of the most differentially expressed genes in the study and has functions involved in not only the organization and degradation of the extracellular matrix, but also cytokine mediated signaling [20]. Decorin can affix to receptors on the surface of macrophages as a way to signal inflammatory cytokines and is involved in a downstream process that reduces available IL-10 [20,37,38]. Since the predicted proteoglycan network (short and extended) is comprised of extracellular glycan (BGN, DCN) and lectin (*VCAN*) binding proteins, the general trend towards downregulation seen for these genes in the HP-PRRSV vs. VR-2332 comparisons could reflect the impact of PRRSV virion replication and N-glycan shielding [14,17,20]. The downregulation of M1 responses despite the presence of mediators and the observation of larger predicted networks may reveal that HP-PRRSV undergoes a more virulent and destructive form of cytopathic replication than VR-2332. The predicted network may represent an alternate pathway for recognition of PRRSV that allows the host to mount an inflammasome response. This pathway may represent a redundancy to counter PRRSV’s ability to affect inflammasome signaling [39] during infection.

Future studies are needed to examine the integration of an antiviral state into the paradigm of conventional activation statuses. This could help to provide a system to comparatively study how monocytic cells are co-opted to have an immunomodulatory effect against different microbes during co-infection, a common observation during PRRSV or other devastating viral infections. However, because of the focus of the current study, the co-infection design was not included.

## Conclusions

The current study showed that the magnitude of differentially expressed gene profiles, clustered mediators, and the predicted network interactions detected in HP-PRRSV rJXwn06 infected mDCs increased compared to VR-2332 infected cells. The study also showed that both viruses inhibit the M1 response activity, which leads to a paucity of pro-inflammatory signaling events to effectively combat viral invasion and replication in mDCs. Many of the genes showing variability in expression, as well as the most common predicted network from the comparisons, were related to cellular structure and the inflammatory immune responses. The results from the current study supply additional insight into the interplay of the viral pathogenicity of PRRSV and its ability to interfere with mDC polarization and activation status.

## Declarations

Mention of trade names or commercial products in this article is solely for the purpose of providing specific information and does not imply recommendation or endorsement by the U.S. Department of Agriculture. USDA is an equal opportunity provider and employer. This study was supported in part by an appointment to the Agricultural Research Service Research Participation Program administered by the Oak Ridge Institute for Science and Education (ORISE) through an interagency agreement between the US Department of Energy (DOE) and the US Department of Agriculture. ORISE is managed by Oak Ridge Associated Universities under DOE contract no. DE-AC05-06OR23100.

## Ethics statement

The animal use protocol was reviewed and approved by the Institutional Animal Care and Use Committee (IACUC) of the National Animal Disease Center-USDA-Agricultural Research Service. Experiments involving animals and viruses were approved by the Kansas State University Institutional Animal Care and Use and Biosafety Committees.

## Supporting information

S1 Table Gene lists from differential gene expression analysis generated from each comparison. Columns show Ensembl ID, rlogFC, and normalized count data for each treatment group and replicate compared. These gene list were used as the query lists for network prediction. The fold changes reported are the difference in expression values for each gene based on the comparison and a threshold of equal to or greater than +/- 2 rlogFc. For comparisons #13, 19, 23, and 26 lists were unable to be generated due to lack of annotation. List does not predicted interactors (protein/chemical) used for each corresponding network.
(XLSX)
